# Potential Role of Vδ2^+^ γδ T Cells in Regulation of Immune Activation in Primary HIV Infection

**DOI:** 10.3389/fimmu.2017.01189

**Published:** 2017-09-25

**Authors:** Nupur Bhatnagar, Pierre-Marie Girard, Moises Lopez-Gonzalez, Céline Didier, Lio Collias, Corinne Jung, Diane Bollens, Claudine Duvivier, Cassandre Von Platen, Daniel Scott-Algara, Laurence Weiss, for the ANRS EP-56 Group, Nadia Valin

**Affiliations:** ^1^Institut Pasteur, Unité Cytokines et Inflammation, Paris, France; ^2^AP-HP, Hôpital Saint-Antoine, Paris, France; ^3^AP-HP, Hôpital Européen Georges Pompidou, Paris, France; ^4^Centre Médical de l’Institut Pasteur, Centre d’Infectiologie Necker Pasteur, Paris, France; ^5^Université Paris Descartes, Sorbonne Paris Cité, Paris, France; ^6^Institut Pasteur, Center for Translational Science, Paris, France

**Keywords:** primary HIV infection, Vδ2^+^ γδ T cells, immune activation, TGF-β, chronic HIV infection

## Abstract

Although conventional regulatory T cells (Tregs) are sufficient in controlling low residual T-cell activation in ART-treated patients, they are not efficient in controlling exaggerated immune activation associated with high levels of HIV replication in primary HIV infection (PHI). Our previous data suggested that double negative (DN) T cells including mainly γδ DN T cells play a role in the control of immune activation in PHI. Since γδ T cells are capable of exerting regulatory functions, we investigated their implication as Tregs in PHI as well as chronic HIV infection (CHI). In a cross-sectional study of 58 HIV-infected patients, in the primary and the chronic phase either ART-treated or untreated (UT), we analyzed phenotype and cytokine production of γδ T cells using flow cytometry. Cytokine production was assessed following *in vitro* stimulation with isopentenyl pyrophosphate or plate-bound anti-CD3/anti-CD28 monoclonal antibodies. We found that the proportion of γδ T cells negatively correlated with CD8 T-cell activation in PHI patients. Furthermore, we found that in these patients, the Vδ2 receptor bearing (Vδ2^+^) γδ T cells were strongly activated, exhibited low terminal differentiation, and produced the anti-inflammatory cytokine, TGF-β. In contrast, in UT-CHI, we observed a remarkable expansion of γδ T cells, where the Vδ2^+^ γδ T cells comprised of an elevated proportion of terminally differentiated cells producing high levels of IFN-γ but very low levels of TGF-β. We also found that this loss of regulatory feature of γδ T cells in CHI was a lasting impairment as we did not find recovery of TGF-β production even in ART-CHI patients successfully treated for more than 5 years. Our data therefore suggest that during the primary HIV infection, Vδ2^+^ γδ T cells may act as Tregs controlling immune activation through production of TGF-β. However, in CHI, γδ T cells transform from an anti-inflammatory into pro-inflammatory cytokine profile and participate in sustenance of immune activation.

## Introduction

Increased T cell turnover, elevated proportions of activated T cells, and augmented levels of pro-inflammatory cytokines and chemokines are hallmarks of chronic immune activation in HIV infection with persistent high viremia (1–3). In the primary HIV infection (PHI), there is prolific viral replication, high levels of immune activation, and induction of HIV-specific CD4 and CD8 T-cell cytotoxic responses (4–6). The rate of CD4 T-cell loss and the subsequent course of infection are determined by the “set point” of CD8 T-cell activation established within the first 6 months following infection (7). While the initial immune response partially controls HIV replication in PHI, it, however, does not eradicate the infection and does not prevent the consequent gradual loss of CD4 T cells and CD8 T-cell functional impairment. In patients treated by ART in chronic HIV infection (CHI) there is rapid decline of activated T cells; however, they rarely reach a normal steady state compared to uninfected individuals (8, 9). Conventional regulatory T cells (Tregs) are known to be capable of controlling inappropriate or exaggerated immune activation mediated either through cellular contact *via* CTLA-4 (10) or through secretion of immunosuppressive cytokines such as IL-10 and TGF-β (11). Although they are competent in controlling low residual T-cell activation in ART-treated patients (12), it was found that they are not sufficient in terms of numbers and/or activity to dampen the exaggerated immune activation that is associated with high levels of HIV replication during PHI (13). Instead, IL-10-producing Foxp3^−^ type I Tregs (Tr1) and double negative (DN) T cells were shown to play a beneficial role in controlling T-cell activation (13, 14). Moreover, in SIV infection, it had been observed that natural hosts had higher proportions of DN T cells than found in pathogenic hosts that were less frequently infected and exhibited polyfunctionality, indicating their critical role in providing help during SIV infection (15, 16).

Double negative T cells are a subclass of T cells with more than 70% of them devoid of CD4 and CD8 (17). They constitute 1–5% of T cells in peripheral blood and lymphoid organs and can express either αβ or γδ T cell receptor. In humans, six Vγ genes (Vγ2,3,4,5,8,9) can combine with three other commonly used Vδ genes (Vδ1,2,3) to create different combinations that allow their preferential homing to specific anatomical localizations. In healthy individuals, Vδ2^+^ cells predominate in peripheral blood, whereas Vδ1^+^ cells and Vδ3^+^ cells are localized in the liver and gut epithelia. Vδ1^+^ cells are also found to be present in thymus, spleen, and dermis (18, 19). Vδ2^+^ cells mainly respond to mycobacterial antigens and tumors. They are also activated by phosphoantigens, such as 4-hydroxy-3-methyl-but-2-enyl pyrophosphate or isopentenyl pyrophosphate (IPP), that get accumulated in virus-infected and cancer cells due to alterations in the mevalonate pathway. Vδ1^+^ and Vδ3^+^ cells participate in defense against viral and fungal infections as well as hematological malignancies (20). In HIV infection, expansion of Vδ1^+^ cells with concomitant depletion of Vδ2^+^ cells in peripheral blood results in an inverted Vδ1^+^/Vδ2^+^ ratio compared to healthy individuals (21, 22). Although not entirely clear, indirect mechanisms involving CCR5/α4β7 signaling as well as direct infection of γδ T cells have been reported to be plausible explanations for Vδ2^+^ cell loss in HIV infection (23–25). Generalized immune activation during UT HIV infection was reported to induce transient expression of CD4 on Vδ2^+^ cells, which enables HIV infection of γδ T cells *in vivo* (25).

As we had previously found that DN T cells including mainly γδ T cells may play a role in controlling high levels of T-cell activation in PHI (13), we put forward the hypothesis that γδ T cells might be involved in the control of immune activation in PHI. Therefore, the primary objective of this study was to characterize phenotype and function of γδ T cells and to investigate their role as potential Tregs in primary HIV infection. We also aimed at analyzing potential changes in the profile of γδ T cells with the stage of infection (primary vs chronic), and their alterations by ART. We thus performed a cross-sectional study including HIV-infected patients in primary and chronic phase with or without antiretroviral treatment.

## Patients, Materials, and Methods

### Study Population

The ANRS-EP56 study comprised three groups of patients. The first group included ART-naïve patients diagnosed with PHI (*n* = 19), symptomatic or not, defined by a negative or weakly positive ELISA, and at least one of the following criteria: incomplete HIV Western Blot, a positive p24 antigenemia and/or detectable plasma HIV-1-RNA. The second and the third groups comprised chronic HIV-infected patients (CHI) either UT (UT-CHI, *n* = 17) or ART-treated (ART-CHI, *n* = 23) with HIV-RNA levels <20 copies/mL since at least 6 months. Non-inclusion criteria included active HCV or HBV infection and ongoing bacterial or opportunistic infection. Patients were prospectively enrolled in the study conducted in three clinical sites in Paris, France between April 2015 and July 2016. Written informed consent was provided by study participants according to French ethical laws. The ethical committee of Ile de France IV approved the study. In addition, a statement of ethical clearance was also received from Institut Pasteur, Paris, which was the sponsor of the study, in charge of administrative and ethical issues. Seventeen healthy donors (HD) were also included in the study. Lymphocyte, CD4, and CD8 counts were measured in all patients routinely using the BD Multitest™ CD3/CD8/CD45/CD4 and BD Trucount™ tubes.

### Blood Sample Processing

Peripheral blood was collected in EDTA-containing tubes. Fresh peripheral blood mononuclear cells (PBMCs) were purified by density gradient centrifugation (Isopaque–Ficoll) within 2–4 h after blood sampling and used the same day for phenotypic and functional analysis.

### Flow Cytometric Analysis

Cells were washed, stained, and analyzed by flow cytometry (LSRII, Becton Dickinson and Gallios, Beckman Coulter). The following monoclonal antibodies (mAbs) were used for staining of surface markers and detection of intracellular cytokines: CD3-ECD (clone UCHT1), γδ-TCR-PC5, γδ-TCR-PE (clone IMMU510), Vδ2-FITC (clone IMMU389), CD27-PE (clone 1A4CD27) (Beckman Coulter); CD4-APC-H7 (clone RPA-T4), CD8-AF700 (clone RPA-T8), IFN-γ-AF700 (clone B27) (Becton Dickinson); CD38-PE-Cy7 (clone HIT2) (eBioscience); HLA-DR-Vioblue (clone AC122), Ki-67-PE-Vio770 (clone REA183) (Miltenyi Biotech); and TGF-β-PE (clone TB21) (IQ Products). FcR-blocking reagent (Miltenyi Biotech) was used to block non-specific Fc-receptor mediated antibody binding. For intracellular staining of Ki-67, IFN-γ, and TGF-β, cells were fixed and permeabilized using the “Foxp3 staining buffer set” (eBioscience) according to the manufacturer’s recommendations. Flow cytometric analysis was performed using FlowJo software (TreeStar).

### Intracellular Cytokine Staining

Following isolation of PBMCs, cells were resuspended in R10 (RPMI 1640 medium supplemented with 10% FCS, penicillin and streptomycin) and stimulated under different conditions. Cells were stimulated with immobilized mAbs against CD3 (1 µg/mL) and soluble CD28 (1 µg/mL) for 24 h at 37°C. Since IFN-γ is an early cytokine, brefeldin A (BFA), 5 µg/mL (Sigma-Aldrich) was added after the first hour of culture with CD3/CD28. Cells were also stimulated with IPP (5 µg/mL) for 4 days at 37°C. In this case, BFA was added to the culture overnight before intracellular staining of TGF-β as it appears later in the time kinetics.

### Statistical Analysis

Data are described as medians and interquartile ranges (IQR). Non-parametric tests were used to avoid the impact of potential outlier values in a small study. Comparisons between groups were performed using the Mann–Whitney test. The Wilcoxon matched-paired test was used to compare the characteristics of the two γδ T-cell subsets. The Spearman’s non-parametric correlation was performed to estimate the association between two continuous variables of interest. *p*-Values below 0.05 were considered as statistically significant.

## Results

### Patients

Clinical characteristics of patients are described in detail in Table 1. Most of the PHI patients were males. At baseline, median age was similar (about 40 years) for PHI and untreated chronic HIV infection (UT-CHI) patients, but higher for ART-CHI patients (52 years). Lymphocyte counts did not differ among the groups of patients. For PHI patients, median duration of time since infection was 36 days (IQR: 32–41). For ART-CHI patients, median duration of ART was 78 months (IQR: 58–221); median duration of time since HIV viral load <20 copies/mL was 48 months (IQR: 24–60) and median nadir CD4 cell count was 281 (IQR: 135–384) cells/μL. Percentage of patients seropositive for CMV in different HIV groups was comparable.

**Table 1:** Healthy donors (HD) and patients’ characteristics.

	HD	Primary HIV infection (PHI)	Untreated chronic HIV infection (UT-CHI)	ART-treated patients (ART-CHI)
Number of individuals	17	19	17	23
Age	45 (37–52)	41 (30–49)	40 (31–44)	52 (40–57)
Sex F/M	4/13	1/18	5/12	5/18
HIV-1 RNA (log_10_/mL)	NA	5.5 (4.7–6.5)	4.2 (4–4.8)	<20
Lymphocyte count (cells/mm^3^)	NA	1,440 (774–2,392)	2,207 (1,504–2,584)	1,891 (1,665–2,380)
CD4 (%)	NA	32 (22–38)	24 (21–38.5)	36 (29–42)
CD4 count (cells/mm^3^)	NA	497 (292–570)	494 (377–815)	723 (602–863)
CD8 (%)	NA	45 (31–58)	48 (40.5–53)	40 (35–43)
CD8 count (cells/mm^3^)	NA	552 (364–1,170)	956 (590–1,229)	704 (542–993)
CD4/CD8 ratio	NA	0.7 (0.4–1.3)	0.6 (0.4–0.9)	0.9 (0.7–1.1)
Individuals seropositive for CMV	60%	79%	88.2%	86.3%

*Data are expressed as median (interquartile ranges)*.

*NA, not available*.

### Distribution of γδ T Cells and Its Subsets in PHI and CHI

**Figure 1:**
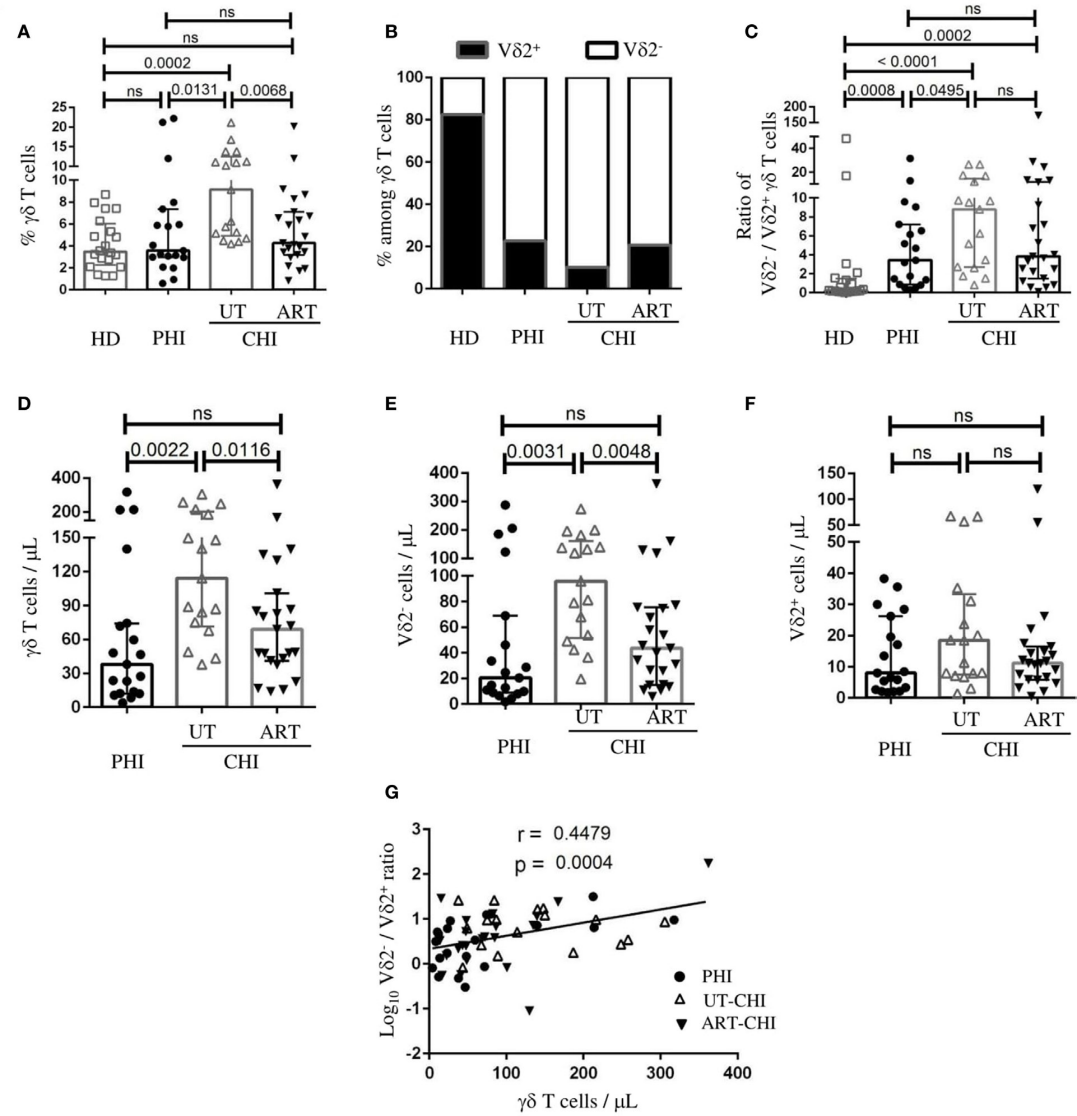
Distribution of γδ T cells and its subsets in peripheral blood. Peripheral blood mononuclear cells were stained *ex vivo* for γδ T cells, as well as its subsets (Vδ2^−^ and Vδ2^+^), and compared between healthy donors (HD), and patients with primary HIV infection (PHI), as well as chronic HIV infection (CHI)—untreated (UT) and treated with ART (ART). **(A)** Frequency of γδ T cells among T cells. **(B)** Graphical representation of frequencies of Vδ2^−^ and Vδ2^+^ cells within γδ T cells. **(C)** Comparison of the ratio of Vδ2^−^/Vδ2^+^ cells among γδ T cells. Comparison of absolute numbers of **(D)** γδ T cells, **(E)** Vδ2^−^ cells, and **(F)** Vδ2^+^ cells between PHI, untreated chronic HIV infection (UT-CHI), and ART-CHI. **(G)** Correlation between absolute count of γδ T cells and log_10_ Vδ2^−^/Vδ2^+^ ratio among γδ T cells in all HIV-infected patients. Data are displayed as median and IQR. Mann–Whitney and Spearman rank correlation tests were performed. Spearman rank correlation coefficient (*r*) is indicated in the panel. *p*-Values are indicated as significant when <0.05; ns, non-significant.

We first analyzed the proportion of γδ T cells and the distribution of its subsets, Vδ2^+^ and Vδ2^−^ cells in PBMCs. Representative flow cytometric staining and gating strategy for γδ T cells is shown in Figure S1 in Supplementary Material. We found that the frequency of γδ T cells was similar between PHI patients and HD [PHI: median 3.5% IQR (3–7.3), HD 3.4% (2.2–6)]. In contrast, a significant increase in the proportion of γδ T cells was observed in the UT-CHI patients compared to HD [UT-CHI 9.1% (5–12.5), *p* = 0.0002] (Figure 1A). We also observed that the distribution of Vδ2^−^/Vδ2^+^ subpopulations within γδ T cells was inversed in HIV-infected patients compared to HD irrespective of the stage of infection (*p* values compared to HD: PHI 0.0008, UT-CHI < 0.0001, ART-CHI 0.0002) (Figures 1B,C). In addition, we found that the ratio of Vδ2^−^/Vδ2^+^ cells was slightly higher in UT-CHI compared to PHI (*p* = 0.04) (Figure 1C). Next, we calculated absolute numbers of γδ T cells and its subsets from total lymphocyte counts and compared it between different groups of HIV-infected patients. Similar to γδ T cell frequency, we found an increase in γδ T cell numbers in UT-CHI compared to PHI [114 (71–201) vs 38 (12–74), *p* = 0.002] whereas both groups had comparable lymphocyte counts (Figure 1D; Table 1). In parallel, the absolute numbers of Vδ2^−^ cells were also elevated in UT-CHI compared to PHI [95 (51–161) vs 20 (9–69), *p* = 0.003] (Figure 1E). In contrast, there was no significant difference in the absolute numbers of Vδ2^+^ cells between the different groups of HIV-infected patients (Figure 1F). In ART-CHI patients, the frequency of γδ T cells [4.2% (3.1–7.1)], and the absolute numbers of both γδ T cells [68 (41–100)] and its subset, Vδ2^−^ cells [43 (15–75)] (Figures 1A,D,E), were lower than in UT-CHI patients. Furthermore, we observed that there was a positive correlation between γδ T cell numbers and the ratio of Vδ2^−^/Vδ2^+^ cells in the whole population of HIV-infected patients (*r* = 0.44, *p* = 0.0004) (Figure 1G).

### γδ T Cell Frequency Is Negatively Correlated with CD8 T-Cell Activation in PHI

**Figure 2:**
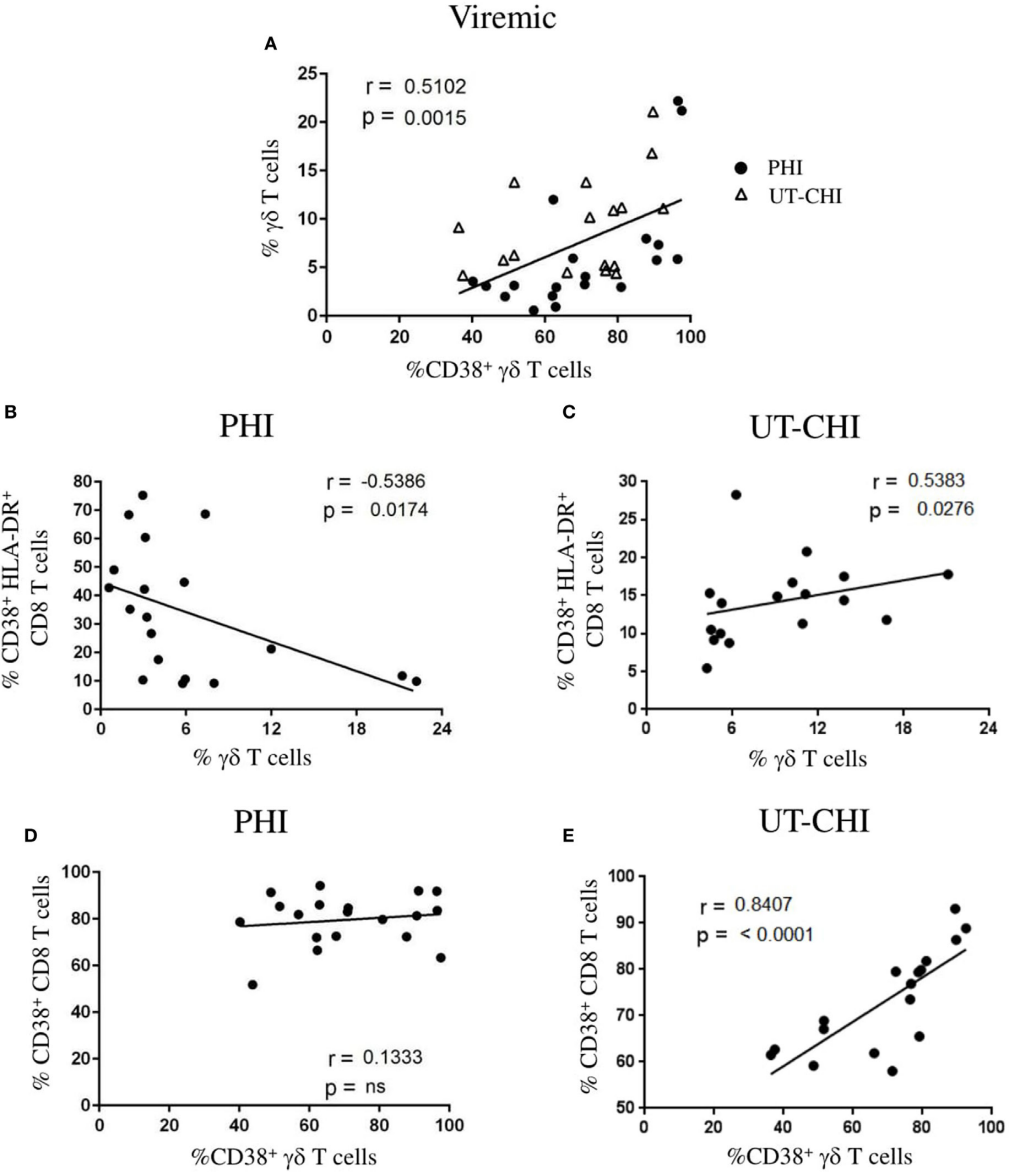
Frequency of γδ T cells is negatively correlated with CD8 T cell activation in primary HIV infection (PHI). **(A)** Relationship between the frequency of γδ T cells and the proportion of CD38^+^ γδ T cells in viremic patients [PHI and untreated chronic HIV infection (UT-CHI)]. **(B,C)** Correlation analysis between the frequency of γδ T cells and the proportion of CD38^+^HLA-DR^+^ CD8 T cells in PHI and UT-CHI. **(D,E)** Correlation between the frequencies of CD38^+^ γδ T cells and CD38^+^CD8 T cells in PHI or UT-CHI. Spearman rank correlation coefficients (*r*) and corresponding *p* values are indicated in each panel; *p*-values are indicated as significant when <0.05; ns, non-significant.

In viremic patients (PHI and UT-CHI), we observed that the frequency of γδ T cells positively correlated with the proportion of activated CD38^+^ γδ T cells (*r* = 0.51, *p* = 0.001) (Figure 2A). To further ascertain the dynamic changes in primary and chronic stages of infection, we analyzed the two groups separately. Interestingly, we found in PHI patients a negative correlation between the proportion of γδ T cells and the proportion of CD38^+^HLA-DR^+^CD8 T cells (*r* = -0.53, *p* = 0.01) (Figure 2B). In contrast, a positive correlation was observed between the proportion of γδ T cells and CD8 T-cell activation in UT-CHI patients (*r* = 0.53, *p* = 0.02) (Figure 2C). Moreover, γδ T-cell activation and CD8 T-cell activation did not correlate in PHI patients (Figure 2D). In contrast, in UT-CHI patients %CD38^+^γδ T cells positively correlated with %CD38^+^CD8 T cells (*r* = 0.84, *p* < 0.0001) (Figure 2E) indicating that generalized chronic immune activation in UT-CHI patients also included activation of γδ T cells.

### Activated Vδ2^+^ γδ T Cells in PHI Exhibit Low Level of Terminal Differentiation Compared to UT-CHI

**Figure 3:**
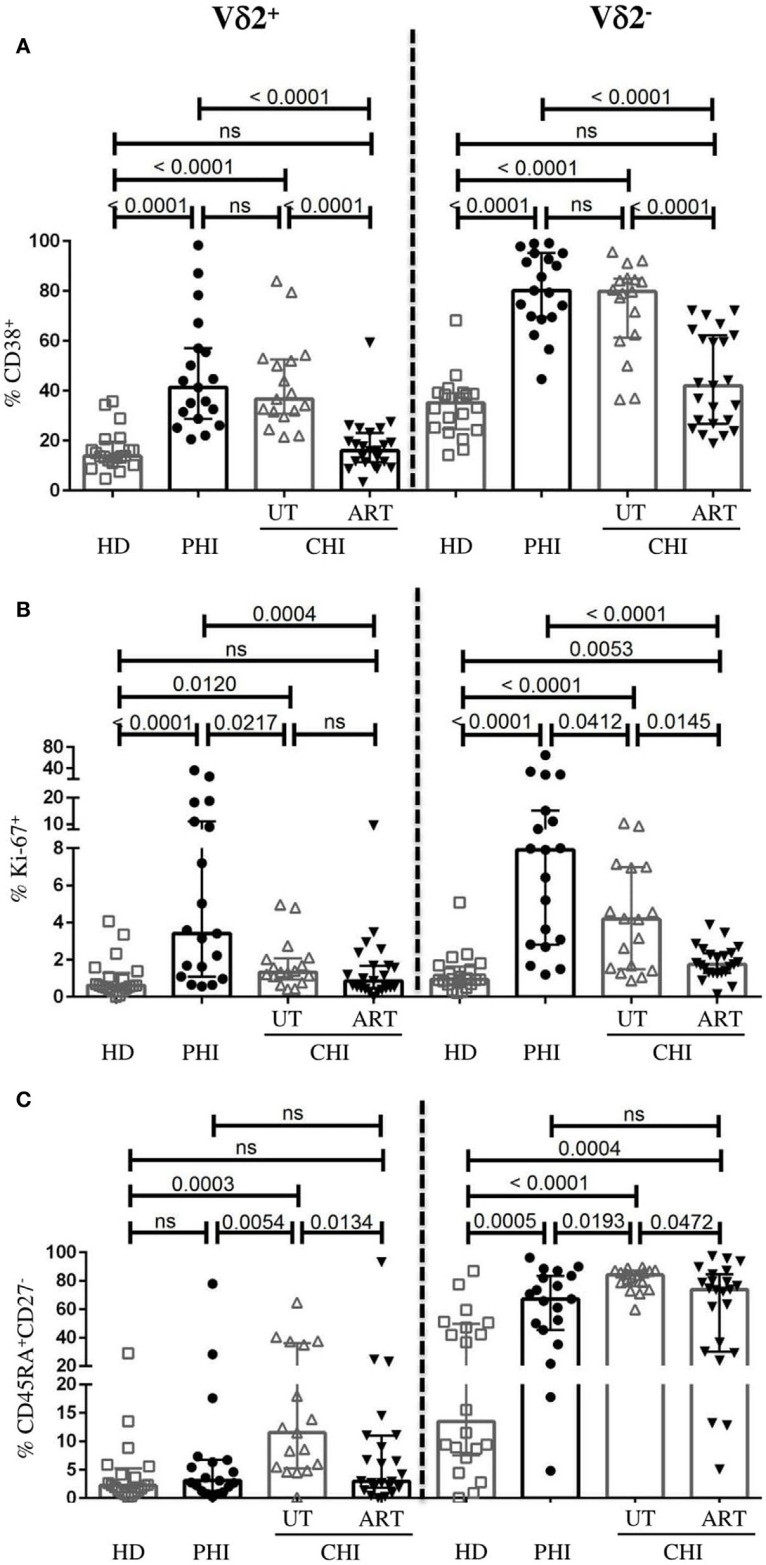
Phenotypic profile of Vδ2^−^ and Vδ2^+^ γδ T cells. Comparison of frequencies of Vδ2^−^ and Vδ2^+^ γδ T cells expressing the following phenotype **(A)** CD38^+^, **(B)** Ki-67^+^, and **(C)** CD45RA^+^CD27^−^ between healthy donors (HD), and patients with primary HIV infection (PHI) as well as chronic HIV infection (CHI)—untreated (UT) and treated with ART (ART). Data are displayed as median and IQR. Mann–Whitney test was performed. *p*-Values are indicated as significant when <0.05; ns, non-significant.

As reported before (26), γδ T cells from HIV-infected patients showed higher levels of CD38. However, the difference between the phenotypic profile of Vδ2^+^ and Vδ2^−^ γδ T cells in the context of HIV infection has not been well characterized. We therefore assessed the activation state, the capacity to proliferate and to differentiate to effector cells of both γδ T-cell subsets in PHI and CHI. In viremic patients (PHI and UT-CHI), we found that both Vδ2^+^ and Vδ2^−^ cells expressed higher levels of CD38 compared to HD (Figure 3A). On the other hand, Ki-67 expression on Vδ2^+^ and Vδ2^−^ cells was strongest in PHI followed by UT-CHI (Figure 3B). The proportion of CD45RA^+^CD27^−^ (terminally differentiated, T_EMRA_) population within the Vδ2^−^ compartment was elevated in both PHI and UT-CHI patients compared to HD. In contrast, among Vδ2^+^ cells, terminal differentiation was found to be significantly lower in PHI compared to UT-CHI; the level was similar to that observed in HD (Figure 3C).

In ART-CHI patients, the proportions of CD38^+^Vδ2^+^ and CD38^+^Vδ2^−^ cells were lower compared to UT-CHI patients with levels comparable to HD (Figure 3A). However, the same was not observed for Ki-67 expression on Vδ2^+^ cells as UT-CHI patients exhibited relatively lower frequency of Ki-67^+^Vδ2^+^ cells compared to HD (Figure 3B). Further, in ART-CHI patients, the frequency of T_EMRA_ cells in the Vδ2^+^ subset and to a lesser extent in the Vδ2^−^ subset was lower than in UT-CHI. The level was comparable to HD for the Vδ2^+^ subset (Figure 3C).

Overall, in all study groups (HD, PHI, UT-CHI, and ART-CHI), the Vδ2^+^ subset comprised a lower proportion of activated (CD38) and terminally differentiated cells (CD45RA^+^CD27^−^) compared to the Vδ2^−^ subset (Figure S2 in Supplementary Material). The frequency of Ki-67^+^ cells was also lower in the Vδ2^+^ subset compared to the Vδ2^−^ subset though the difference was not significant in all study groups.

To sum up, Vδ2^+^ cells in PHI patients were activated to similar levels as in UT-CHI patients but exhibited significantly lower levels of terminal differentiation.

### Anti-inflammatory Cytokine Profile of Vδ2^+^ γδ T Cells in PHI

**Figure 4:**
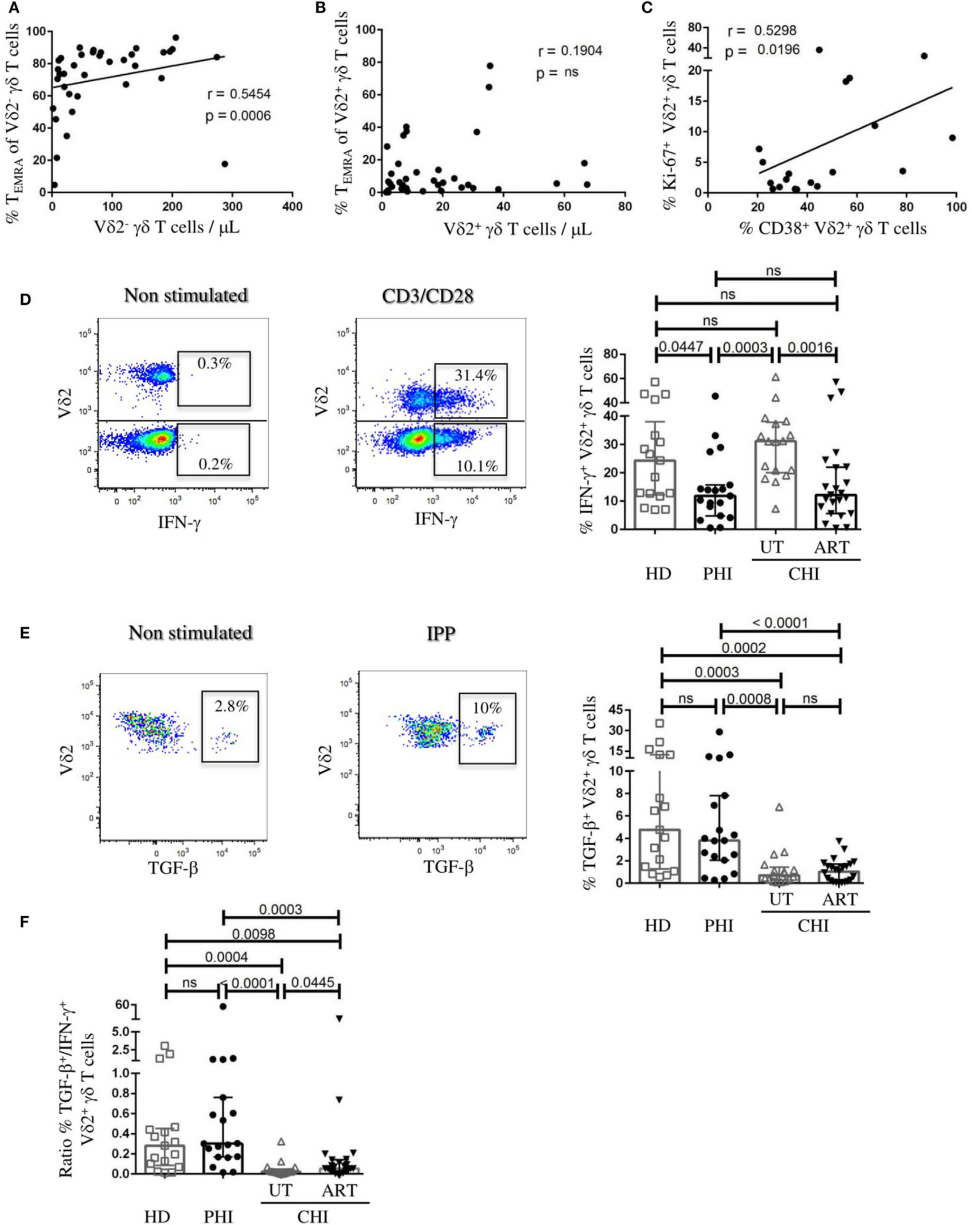
Vδ2^+^ γδ T cells exhibit anti-inflammatory cytokine profile in primary HIV infection (PHI). **(A)** Correlation analyses in viremic patients **(A)** between absolute numbers of Vδ2^−^ γδ T cells and the proportion of CD45RA^+^CD27^−^ (T_EMRA_) Vδ2^−^ γδ T cells, and **(B)** between Vδ2^+^ γδ T cells and the proportion of CD45RA^+^CD27^−^ (T_EMRA_) Vδ2^+^ γδ T cells. **(C)** Relationship between the frequency of Ki-67^+^Vδ2^+^ γδ T cells and the proportion of CD38^+^Vδ2^+^ γδ T cells in PHI patients. **(D)** Representative FACS staining of IFN-γ production by Vδ2^+^ and Vδ2^−^ γδ T cells from a UT-CHI patient with or without CD3/CD28 stimulation for 24 h. Comparison of frequencies of IFN-γ^+^Vδ2^+^ γδ T cells between healthy donors (HD), and patients with primary HIV infection (PHI) as well as chronic HIV infection (CHI)—untreated (UT) and treated with ART (ART). **(E)** Representative FACS staining of TGF-β production by Vδ2^+^ γδ T cells from a PHI patient with or without 4 days of isopentenyl pyrophosphate (IPP) stimulation. Comparison of frequencies of TGF-β^+^Vδ2^+^ γδ T cells between HD, PHI, UT-CHI, and ART-CHI. **(F)** Comparison of the ratio of frequencies of TGF-β^+^/IFN-γ^+^Vδ2^+^ γδ T cells between HD, PHI, UT-CHI, and ART-CHI. Data are displayed as median and IQR. Mann–Whitney and Spearman rank correlation tests were performed. Spearman rank correlation coefficients (*r*) are indicated in the panels. *p*-Values are indicated as significant when <0.05; ns, non-significant.

We observed a strong positive relationship between the frequency of Vδ2^−^ T_EMRA_ cells and the number of Vδ2^−^ cells in viremic patients (*r* = 0.54, *p* = 0.0006) (Figure 4A). In contrast, we did not find such association for Vδ2^+^ cells (Figure 4B). Furthermore, in PHI patients there was positive correlation between %CD38^+^Vδ2^+^ cells and %Ki-67^+^Vδ2^+^ cells indicating that Vδ2^+^ cells were not just activated but were also undergoing cellular proliferation (*r* = 0.52, *p* = 0.02) (Figure 4C). Following our observation that in PHI, frequency of γδ T cells negatively correlated with CD8 T-cell activation and that the Vδ2^+^ subset exhibited low level of terminal differentiation, we wanted to investigate their functional competence. The left panels of Figures 4D,E illustrate representative flow cytometry staining of IFN-γ and TGF-β production following anti-CD3/-CD28 and IPP stimulation, respectively. We found in PHI a lower proportion of Vδ2^+^ cells producing IFN-γ compared to UT-CHI [PHI 11.8% (4.8–15.7), UT-CHI 31.1% (20–37.7), *p* = 0.0003] (Figure 4D). Conversely, Vδ2^+^ cells produced more TGF-β in PHI patients than in UT-CHI patients [PHI 3.8% (2.0–7.8), UT-CHI 0.7% (0.2–1.4), *p* = 0.0008] (Figure 4E). We also observed that the proportion of TGF-β^+^Vδ2^+^ cells negatively correlated with the proportion of Vδ2^+^ T_EMRA_ cells in viremic patients (data not shown). Interestingly, the ratio of the frequencies of TGF-β^+^/IFN-γ^+^Vδ2^+^ cells was higher in PHI compared to UT-CHI [PHI 0.3 (0.1–0.7), UT-CHI 0.02 (0.01–0.05), *p* < 0.0001]. The ratio was similar between PHI and HD (Figure 4F).

In ART-treated patients, there was lower proportion of IFN-γ^+^Vδ2^+^ cells compared to UT-CHI patients [ART-CHI 12.1% (5.6–22), *p* = 0.001] (Figure 4D), but little or no production of TGF-β [ART-CHI 1% (0.1–1.7)] as observed in UT-CHI patients (Figure 4E). The ratio of the frequencies of TGF-β^+^/IFN-γ^+^Vδ2^+^ cells was low and similar in UT-CHI and ART-CHI patients (Figure 4F).

We found that there was no difference in IFN-γ production capacity of Vδ2^−^ cells between different study groups (Figure S3 in Supplementary Material). Further, unlike Vδ2^+^ cells, Vδ2^−^ cells did not produce any TGF-β (data not shown).

To conclude, Vδ2^+^ cells in PHI patients exhibited an anti-inflammatory cytokine production profile with high TGF-β and low IFN-γ production.

## Discussion

In this study, we found that the dynamics in γδ T cells were distinct in different clinical stages of HIV infection—primary vs chronic. First, we observed that the γδ T cell frequency was similar between PHI patients and HD; nevertheless, UT-CHI patients exhibited a higher proportion of γδ T cells. This observation was in contrast with one study that showed no difference in the proportion and absolute count of γδ T cells between healthy controls, acute HIV infection, and slow and fast progressors (26). However, earlier studies have reported an increase in γδ T cells in HIV infection as well as an inverted Vδ1^+^/Vδ2^+^ ratio, a consequence of expansion of Vδ1^+^ cells and/or depletion of Vδ2^+^ cells (21, 22, 27). In accordance with them, we also found that there was skewed Vδ2^−^/Vδ2^+^ ratio, which positively correlated with γδ T cell numbers in HIV-infected patients including PHI and CHI with or without ART. Second, the proportion of γδ T cells negatively correlated with CD8 T-cell activation in PHI, which was in contrast with UT-CHI where we observed a positive correlation. We had previously reported that in PHI patients, the proportion of DN T cells negatively correlated with the proportion of CD38^+^HLA-DR^+^CD8 T cells within the first weeks of acute infection (13). In addition, the frequency of DN T cells at baseline predicted the level of CD8 T-cell activation at month 6 [i.e., the immune set point (7)]. Fifty percent of DN T cells expressed the γδ TCR. Earlier studies have demonstrated that γδ T cells are able to suppress innate and adaptive immune responses (28–30). Third, we found that Vδ2^+^ γδ T cells comprised a significantly lower proportion of terminally differentiated cells in PHI compared to UT-CHI. Furthermore in PHI patients, Vδ2^+^ γδ T cells displayed an anti-inflammatory cytokine profile whereas in UT-CHI patients Vδ2^+^ cells exhibited pro-inflammatory cytokine characteristics. Taken together, our findings suggest a potential role of γδ T cells as immune regulators in PHI.

Alterations in the phenotype and function of the γδ T cell compartment have been observed in several viral infections (31). In CMV infection, it has been shown that Vδ2^+^ and Vδ2^−^ cells display distinct evolution and phenotypic pattern. As opposed to Vδ2^+^ subset, the Vδ2^−^ subset was found to express higher levels of CD38 in patients with active CMV infection (32). During primary CMV infection, Roux et al. reported that activated Vδ2^−^ cells directly correlated with CD8 T-cell activation (32). In our study, we observed that there was a positive association between activated γδ T cells and activated CD8 T cells in UT-CHI but not in PHI clearly indicating that during CHI, immune activation encompasses all T-cell subsets. We also found that although level of CD38 expression on Vδ2^+^ cells was similar between PHI and UT-CHI, the %Ki-67^+^Vδ2^+^ cells was significantly higher in PHI compared to UT-CHI indicating active proliferation of these cells during acute infection. A positive correlation of %CD38^+^Vδ2^+^ cells and %Ki-67^+^Vδ2^+^ cells supported this observation. It was recently demonstrated that immune activation can render Vδ2^+^ cells susceptible to HIV infection and serve as latent reservoir for HIV (25). We found that there was a high ratio of Vδ2^−^/Vδ2^+^ cells in all groups of HIV-infected patients compared to HD. Noteworthy, the absolute numbers of Vδ2^+^ cells remained unperturbed between different groups of HIV patients despite strong activation and cellular proliferation of Vδ2^+^ cells in PHI. We were therefore intrigued by the nature of Vδ2^+^ cells, which motivated us to analyze them further. It is well known that as cells proceed toward terminal differentiation they lose their proliferative potential and effector functions and progress to exhaustion. We observed that such events happened to occur in the Vδ2^−^ subset as in viremic patients, the number of Vδ2^−^ cells positively correlated with the proportion of terminally differentiated Vδ2^−^ cells. In contrast, no such correlation was observed for Vδ2^+^ cells suggesting that Vδ2^+^ subset of γδ T cells could be involved in immune regulation in PHI. Both mucosal and circulating Vδ1^+^ cells in CHI have been shown to have high frequency of T_EMRA_ cells compared to PHI (27). Furthermore, in CMV infection, it was observed that the Vδ2^−^ subset exhibited predominance of terminally differentiated cells compared to Vδ2^+^ subset (32, 33). It is suggested that differentiation phenotype described in γδ T-cell subsets could be due to the type of stimulus or pathogen they react to (phosphoantigens for Vδ2^+^ γδ T cells) (32). Therefore, to specifically activate Vδ2^+^ cells and investigate their functional capacity, we used IPP as the stimulus. In addition, we used mAbs against CD3 and CD28 to stimulate both Vδ2^+^ and Vδ2^−^ cells and to assess and compare their cytokine production in PHI and CHI. We found that Vδ2^+^ cells were able to produce both the anti-inflammatory cytokine TGF-β as well as the pro-inflammatory cytokine IFN-γ. Interestingly, there was a high %TGF-β^+^ but low frequency of IFN-γ producers among Vδ2^+^ cells in PHI patients compared to UT-CHI patients. In viremic patients, the %TGF-β^+^Vδ2^+^ cells negatively correlated with the %Vδ2^+^ T_EMRA_ cells indicating that higher was the terminal differentiation of Vδ2^+^ cells the lower was their ability to produce TGF-β. In addition, we observed that the ratio of TGF-β^+^/IFN-γ^+^Vδ2^+^ cells was significantly higher in PHI compared to UT-CHI indicating the conversion of Vδ2^+^ cells from an anti-inflammatory cytokine profile in PHI to pro-inflammatory profile in UT-CHI.

Both Vδ1^+^ and Vδ2^+^ cells have been shown to be able to produce TGF-β upon *in vitro* stimulation with anti-human TCR Vδ1 and CD3/CD28, respectively (29, 34). Vδ2^+^ cells can also suppress T cell proliferation and IL-2 production *via* interaction of CD86 with CTLA-4 on activated CD4 T cells (30). It is known that IFN-γ along with other inflammatory cytokines contributes to the establishment of chronic immune activation during HIV infection (35). On the other hand, TGF-β has been shown to regulate T cell responses by suppressing their proliferation and IL-2 production (36). We previously showed that systemic immune activation in primary HIV infection is primarily driven by the virus and innate immune responses. Microbial translocation probably does not occur at the time of early PHI (37, 38) despite a loss of intestinal mucosal integrity (39, 40). Our findings in this study suggest that, γδ T cells, particularly Vδ2^+^ cells, might play a role in the control of excessive T-cell activation in PHI through production of TGF-β. In contrast, in UT-CHI, the regulatory capacity of Vδ2^+^ cells is overridden by the presence of viral replication, pro-inflammatory cytokines and other soluble factors that are driving systemic immune activation. The fact that we observed a negative correlation of γδ T cell frequency with CD8 T-cell activation in PHI, but found no such association with either the Vδ2^+^ or Vδ2^−^ subset when assessed separately (data not shown), suggests that probably both subsets work in partnership as immune regulators in PHI. It has been demonstrated that Vδ1^+^ cells can also produce TGF-β (34). We therefore cannot rule out the possibility that Vδ2^−^ cells producing TGF-β may also play a role in the control of immune activation in PHI.

Although ART results in a dramatic decrease in HIV viremia and systemic immune activation, the level of residual immune activation does not necessarily reach that of HIV-uninfected individuals, especially in patients treated late in the chronic phase of infection (8, 9). In our study, we found that in ART-CHI patients, the proportion of activated as well as terminally differentiated Vδ2^+^ cells was reduced to the level of HD. It has been shown that despite the reconstitution of TCR repertoire, the numbers and functions of Vδ2^+^ cells are not totally restored even after prolonged ART (41). Consistent with these reports we found that although the frequency of γδ T cells was reinstated in ART-CHI patients compared to UT-CHI patients, the ratio of Vδ2^−^/Vδ2^+^ cells remained impaired. IFN-γ levels in serum are shown to decline in HIV-infected patients after initiation of ART (35). We also found that the proportion of IFN-γ^+^Vδ2^+^ cells was reduced in ART-CHI compared to UT-CHI patients. In contrast, the frequency of TGF-β producing Vδ2^+^ cells did not recover in ART-CHI patients. Moreover, the ratio of TGF-β^+^/IFN-γ^+^Vδ2^+^ cells was similar between UT-CHI and ART-CHI patients indicating that there was a lasting damage to the regulatory capacity of Vδ2^+^ cells in CHI patients despite effective ART.

There were some limitations in our study. The cross-sectional nature of this study provided valuable insight into the phenotypic characteristics and functional capacities of γδ T cells in different stages of infection—primary, UT chronic and ART-treated chronic. However, a longitudinal design would have allowed individual follow up on the evolution of γδ T cell features during the course of infection. The benefit of a cross-sectional design was that we have analyzed patients who have initiated ART during the chronic phase. A longitudinal design would have been limited to the analysis of patients who had been treated early during chronic infection, the current situation for about 50% of the patients in the real life in Europe (42). Our study focused on analysis of γδ T cells in peripheral blood. We had no access to tissue samples, particularly from the gastro-intestinal tract. Analyses of tissues would have allowed a better understanding of the dynamic changes in the distribution of γδ T cell subsets and their functions.

In summary, we report on the potential role of γδ T cells in the control of immune activation in PHI. We showed that the proportion of γδ T cells negatively correlated with CD8 T-cell activation in PHI patients, and that the Vδ2^+^ subset was probably the predominant player mediating its effect *via* production of the anti-inflammatory cytokine, TGF-β. Furthermore, we found that there was a sustainable loss in the immune regulatory capacity of Vδ2^+^ γδ T cells in CHI as there was no recovery observed in their function even in ART-treated patients with median duration of suppressed viral load of more than 5 years. Thus based on our data, we believe that therapies aiming to restore the functional properties of Vδ2^+^ γδ T-cell subset in CHI should be considered.

## Ethics Statement

This study was carried out in accordance with the recommendations of “French ethical laws” with written informed consent from all patients. All patients gave written informed consent in accordance with the Declaration of Helsinki. The protocol was approved by “The ethical committee of Ile de France IV.”

## Author Contributions

LW designed and supervised the study. NB and LW contributed to experimental design and wrote the manuscript. NB analyzed the data. NB, ML-G, and CDi performed experiments. P-MG, LC, DB, and CDu included patients. CJ collected and analyzed clinical data. CP implemented and managed the project. P-MG and DS-A reviewed the manuscript.

## Conflict of Interest Statement

The authors declare that the research was conducted in the absence of any commercial or financial relationships that could be construed as a potential conflict of interest.

## ANRS-EP56 Group

**Nadia Valin**, Hôpital Saint Antoine, Paris; **Laurent Fonquernie**, Hôpital Saint Antoine, Paris; **Marina Karmochkine**, Hôpital Européen Georges Pompidou, Paris; **Philippe Castiel**, Hôpital Européen Georges Pompidou, Paris; **Anne Dumont**, Service des recherches cliniques sur les hépatites virales et le VIH-des recherches fondamentales sur les hépatites virales, ANRS-INSERM, Paris; **Lucie Marchand**, Service des recherches cliniques sur les hépatites virales et le VIH-des recherches fondamentales sur les hépatites virales, ANRS-INSERM, Paris.

## References

[1] Hellerstein M Hanley MB Cesar D Siler S Papageorgopoulos C Wieder E Directly measured kinetics of circulating T lymphocytes in normal and HIV-1-infected humans. Nat Med (1999) 5(1):83–9. 10.1038/4772 9883844

[2] Hazenberg MD Stuart JW Otto SA Borleffs JC Boucher CA de Boer RJ T-cell division in human immunodeficiency virus (HIV)-1 infection is mainly due to immune activation: a longitudinal analysis in patients before and during highly active antiretroviral therapy (HAART). Blood (2000) 95(1):249–55. 10607709

[3] Valdez H Lederman MM Cytokines and cytokine therapies in HIV infection. AIDS Clin Rev (1997):187–228. 9305449

[4] Borrow P Lewicki H Hahn BH Shaw GM Oldstone MB. Virus-specific CD8+ cytotoxic T-lymphocyte activity associated with control of viremia in primary human immunodeficiency virus type 1 infection. J Virol (1994) 68(9):6103–10. 8057491 10.1128/jvi.68.9.6103-6110.1994 PMC237022

[5] Kahn JO Walker BD Acute human immunodeficiency virus type 1 infection. N Engl J Med (1998) 339(1):33–9. 10.1056/NEJM199807023390107 9647878

[6] Cohen MS Shaw GM McMichael AJ Haynes BF Acute HIV-1 infection. N Engl J Med (2011) 364(20):1943–54. 10.1056/NEJMra1011874 21591946 PMC3771113

[7] Deeks SG Kitchen CM Liu L Guo H Gascon R Narváez AB Immune activation set point during early HIV infection predicts subsequent CD4+ T-cell changes independent of viral load. Blood (2004) 104(4):942–7. 10.1182/blood-2003-09-3333 15117761

[8] Hunt PW Martin JN Sinclair E Bredt B Hagos E Lampiris H T cell activation is associated with lower CD4+ T cell gains in human immunodeficiency virus-infected patients with sustained viral suppression during antiretroviral therapy. J Infect Dis (2003) 187(10):1534–43. 10.1086/374786 12721933

[9] Robbins GK Spritzler JG Chan ES Asmuth DM Gandhi RT Rodriguez BA Incomplete reconstitution of T cell subsets on combination antiretroviral therapy in the AIDS Clinical Trials Group protocol 384. Clin Infect Dis (2009) 48(3):350–61. 10.1086/595888 19123865 PMC2676920

[10] Read S Malmström V Powrie F. Cytotoxic T lymphocyte-associated antigen 4 plays an essential role in the function of CD25(+)CD4(+) regulatory cells that control intestinal inflammation. J Exp Med (2000) 192(2):295–302. 10.1084/jem.192.2.295 10899916 PMC2193261

[11] von Boehmer H. Mechanisms of suppression by suppressor T cells. Nat Immunol (2005) 6(4):338–44. 10.1038/ni1180 15785759

[12] Weiss L Piketty C Assoumou L Didier C Caccavelli L Donkova-Petrini V Relationship between regulatory T cells and immune activation in human immunodeficiency virus-infected patients interrupting antiretroviral therapy. PLoS One (2010) 5(7):e11659. 10.1371/journal.pone.0011659 20657770 PMC2908121

[13] Petitjean G Chevalier MF Tibaoui F Didier C Manea ME Liovat AS Level of double negative T cells, which produce TGF-β and IL-10, predicts CD8 T-cell activation in primary HIV-1 infection. AIDS (2012) 26(2):139–48. 10.1097/QAD.0b013e32834e1484 22045342

[14] Chevalier MF Didier C Petitjean G Karmochkine M Girard PM Barré-Sinoussi F Phenotype alterations in regulatory T-cell subsets in primary HIV infection and identification of Tr1-like cells as the main interleukin 10-producing CD4+ T cells. J Infect Dis (2015) 211(5):769–79. 10.1093/infdis/jiu549 25281758

[15] Vinton C Klatt NR Harris LD Briant JA Sanders-Beer BE Herbert R CD4-like immunological function by CD4- T cells in multiple natural hosts of simian immunodeficiency virus. J Virol (2011) 85(17):8702–8. 10.1128/JVI.00332-11 21715501 PMC3165829

[16] Sundaravaradan V Saleem R Micci L Gasper MA Ortiz AM Else J Multifunctional double-negative T cells in sooty mangabeys mediate T-helper functions irrespective of SIV infection. PLoS Pathog (2013) 9(6):e1003441. 10.1371/journal.ppat.1003441 23825945 PMC3694849

[17] Kalyan S Kabelitz D Defining the nature of human γδ T cells: a biographical sketch of the highly empathetic. Cell Mol Immunol (2013) 10(1):21–9. 10.1038/cmi.2012.44 23085947 PMC4003173

[18] Kabelitz D He W. The multifunctionality of human Vγ9Vδ2 γδ T cells: clonal plasticity or distinct subsets? Scand J Immunol (2012) 76(3):213–22. 10.1111/j.1365-3083.2012.02727.x 22670577

[19] Bonneville M O’Brien RL Born WK. Gammadelta T cell effector functions: a blend of innate programming and acquired plasticity. Nat Rev Immunol (2010) 10(7):467–78. 10.1038/nri2781 20539306

[20] Poggi A Zocchi MR γδ T Lymphocytes as a first line of immune defense: old and new ways of antigen recognition and implications for cancer immunotherapy. Front Immunol (2014) 5:575 10.3389/fimmu.2014.00575 25426121 PMC4226920

[21] Boullier S Cochet M Poccia F Gougeon ML. CDR3-independent gamma delta V delta 1+ T cell expansion in the peripheral blood of HIV-infected persons. J Immunol (1995) 154(3):1418–31. 7822807

[22] Poccia F Boullier S Lecoeur H Cochet M Poquet Y Colizzi V Peripheral V gamma 9/V delta 2 T cell deletion and anergy to nonpeptidic mycobacterial antigens in asymptomatic HIV-1-infected persons. J Immunol (1996) 157(1):449–61. 8683151

[23] Li H Pauza CD. HIV envelope-mediated, CCR5/α4β7-dependent killing of CD4-negative γδ T cells which are lost during progression to AIDS. Blood (2011) 118(22):5824–31. 10.1182/blood-2011-05-356535 21926353 PMC3228498

[24] Imlach S Leen C Bell JE Simmonds P. Phenotypic analysis of peripheral blood gammadelta T lymphocytes and their targeting by human immunodeficiency virus type 1 in vivo. Virology (2003) 305(2):415–27. 10.1006/viro.2002.1759 12573587

[25] Soriano-Sarabia N Archin NM Bateson R Dahl NP Crooks AM Kuruc JD Peripheral Vγ9Vδ2 T cells are a novel reservoir of latent HIV infection. PLoS Pathog (2015) 11(10):e1005201. 10.1371/journal.ppat.1005201 26473478 PMC4608739

[26] Li Z Jiao Y Hu Y Cui L Chen D Wu H Distortion of memory Vδ2 γδ T cells contributes to immune dysfunction in chronic HIV infection. Cell Mol Immunol (2015) 12(5):604–14. 10.1038/cmi.2014.77 25220734 PMC4579648

[27] Cimini E Agrati C D’Offizi G Vlassi C Casetti R Sacchi A Primary and chronic HIV infection differently modulates mucosal Vδ1 and Vδ2 T-cells differentiation profile and effector functions. PLoS One (2015) 10(6):e0129771 10.1371/journal.pone.0129771 26086523 PMC4472518

[28] Ye J Ma C Hsueh EC Eickhoff CS Zhang Y Varvares MA Tumor-derived γδ regulatory T cells suppress innate and adaptive immunity through the induction of immunosenescence. J Immunol (2013) 190(5):2403–14. 10.4049/jimmunol.1202369 23355732 PMC3578061

[29] Kühl AA Pawlowski NN Grollich K Blessenohl M Westermann J Zeitz M Human peripheral gammadelta T cells possess regulatory potential. Immunology (2009) 128(4):580–8. 10.1111/j.1365-2567.2009.03162.x 19807790 PMC2792142

[30] Peters C Oberg HH Kabelitz D Wesch D Phenotype and regulation of immunosuppressive Vδ2-expressing γδ T cells. Cell Mol Life Sci (2014) 71(10):1943–60. 10.1007/s00018-013-1467-1 24091816 PMC3997799

[31] Zheng J Liu Y Lau YL Tu W. γδ-T cells: an unpolished sword in human anti-infection immunity. Cell Mol Immunol (2013) 10(1):50–7. 10.1038/cmi.2012.43 23064104 PMC4003172

[32] Roux A Mourin G Larsen M Fastenackels S Urrutia A Gorochov G Differential impact of age and cytomegalovirus infection on the γδ T cell compartment. J Immunol (2013) 191(3):1300–6. 10.4049/jimmunol.1202940 23817410

[33] Pitard V Roumanes D Lafarge X Couzi L Garrigue I Lafon ME Long-term expansion of effector/memory Vdelta2-gammadelta T cells is a specific blood signature of CMV infection. Blood (2008) 112(4):1317–24. 10.1182/blood-2008-01-136713 18539896 PMC2515135

[34] Hua F Kang N Gao YA Cui LX Ba DN He W. Potential regulatory role of in vitro-expanded Vδ1 T cells from human peripheral blood. Immunol Res (2013) 56(1):172–80. 10.1007/s12026-013-8390-2 23532670

[35] Roff SR Noon-Song EN Yamamoto JK. The significance of interferon-γ in HIV-1 pathogenesis, therapy, and prophylaxis. Front Immunol (2014) 4:498. 10.3389/fimmu.2013.00498 24454311 PMC3888948

[36] Li MO Wan YY Sanjabi S Robertson AK Flavell RA. Transforming growth factor-beta regulation of immune responses. Annu Rev Immunol (2006) 24:99–146. 10.1146/annurev.immunol.24.021605.090737 16551245

[37] Chevalier MF Petitjean G Dunyach-Rémy C Didier C Girard PM Manea ME The Th17/Treg ratio, IL-1RA and sCD14 levels in primary HIV infection predict the T-cell activation set point in the absence of systemic microbial translocation. PLoS Pathog (2013) 9(6):e1003453. 10.1371/journal.ppat.1003453 23818854 PMC3688532

[38] Chevalier MF Didier C Girard PM Manea ME Campa P Barré-Sinoussi F CD4 T-cell responses in primary HIV infection: interrelationship with immune activation and virus burden. Front Immunol (2016) 7:395. 10.3389/fimmu.2016.00395 27746782 PMC5040706

[39] Jenabian MA El-Far M Vyboh K Kema I Costiniuk CT Thomas R Immunosuppressive tryptophan catabolism and gut mucosal dysfunction following early HIV infection. J Infect Dis (2015) 212(3):355–66. 10.1093/infdis/jiv037 25616404

[40] Sereti I Krebs SJ Phanuphak N Fletcher JL Slike B Pinyakorn S Persistent, albeit reduced, chronic inflammation in persons starting antiretroviral therapy in acute HIV infection. Clin Infect Dis (2017) 64(2):124–31. 10.1093/cid/ciw683 27737952 PMC5215214

[41] Pauza CD Poonia B Li H Cairo C Chaudhry D γδ T cells in HIV disease: past, present, and future. Front Immunol (2014) 5:687 10.3389/fimmu.2014.00687 25688241 PMC4311680

[42] Mocroft A Lundgren JD Sabin ML Monforte A Brockmeyer N Casabona J Risk factors and outcomes for late presentation for HIV-positive persons in Europe: results from the Collaboration of Observational HIV Epidemiological Research Europe Study (COHERE). PLoS Med (2013) 10(9):e1001510. 10.1371/journal.pmed.1001510 24137103 PMC3796947

