# Supplementary figures and images for: Potential Role of Vδ2+ γδ T Cells in Regulation of Immune Activation in Primary HIV Infection
Source: Front Immunol. 2017 Sep 25;8:1189. doi: 10.3389/fimmu.2017.01189 (PMC5622291; doi:10.3389/fimmu.2017.01189)

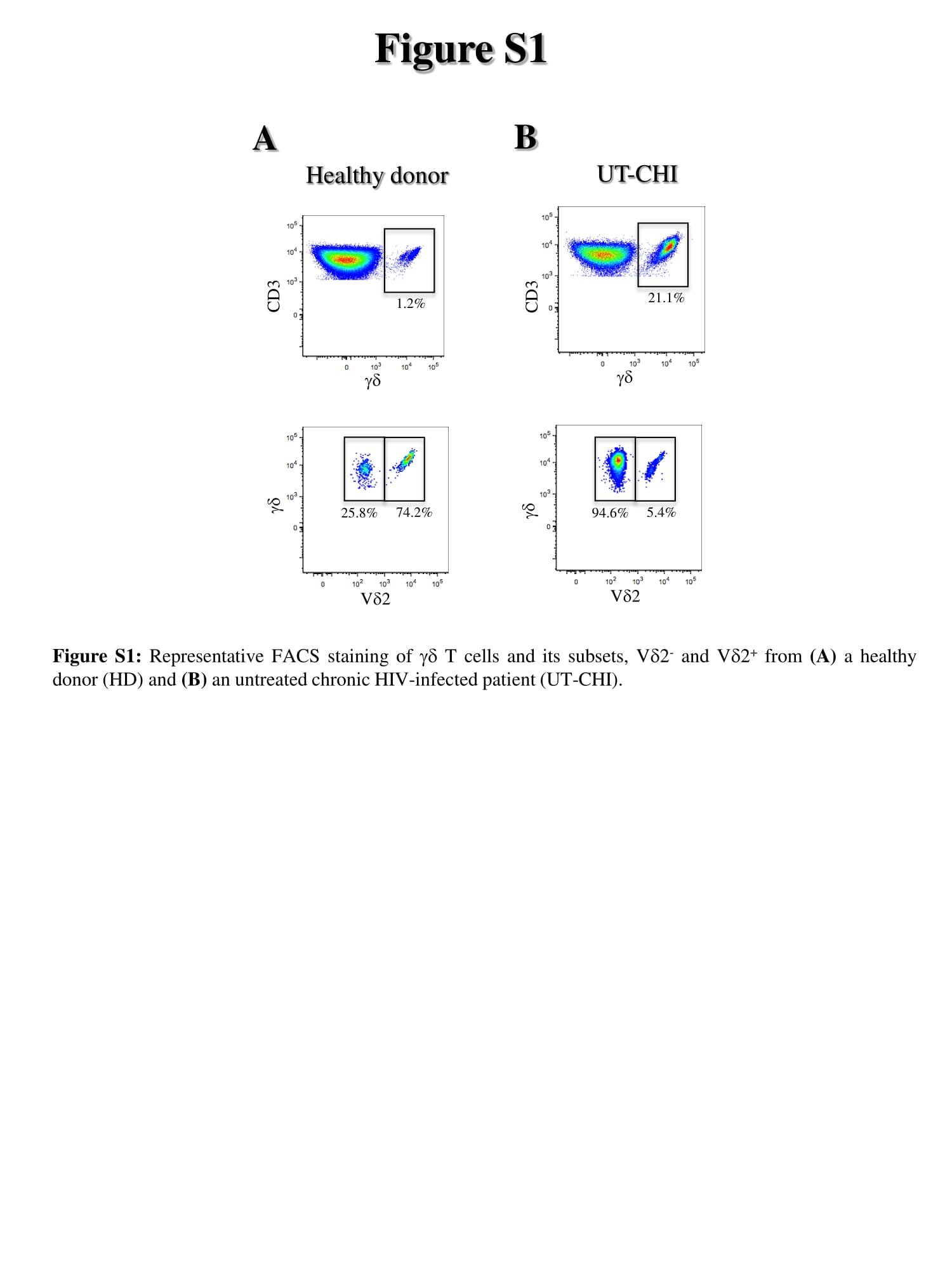

Supplement: Supplementary file 1 [file image_1.jpeg]

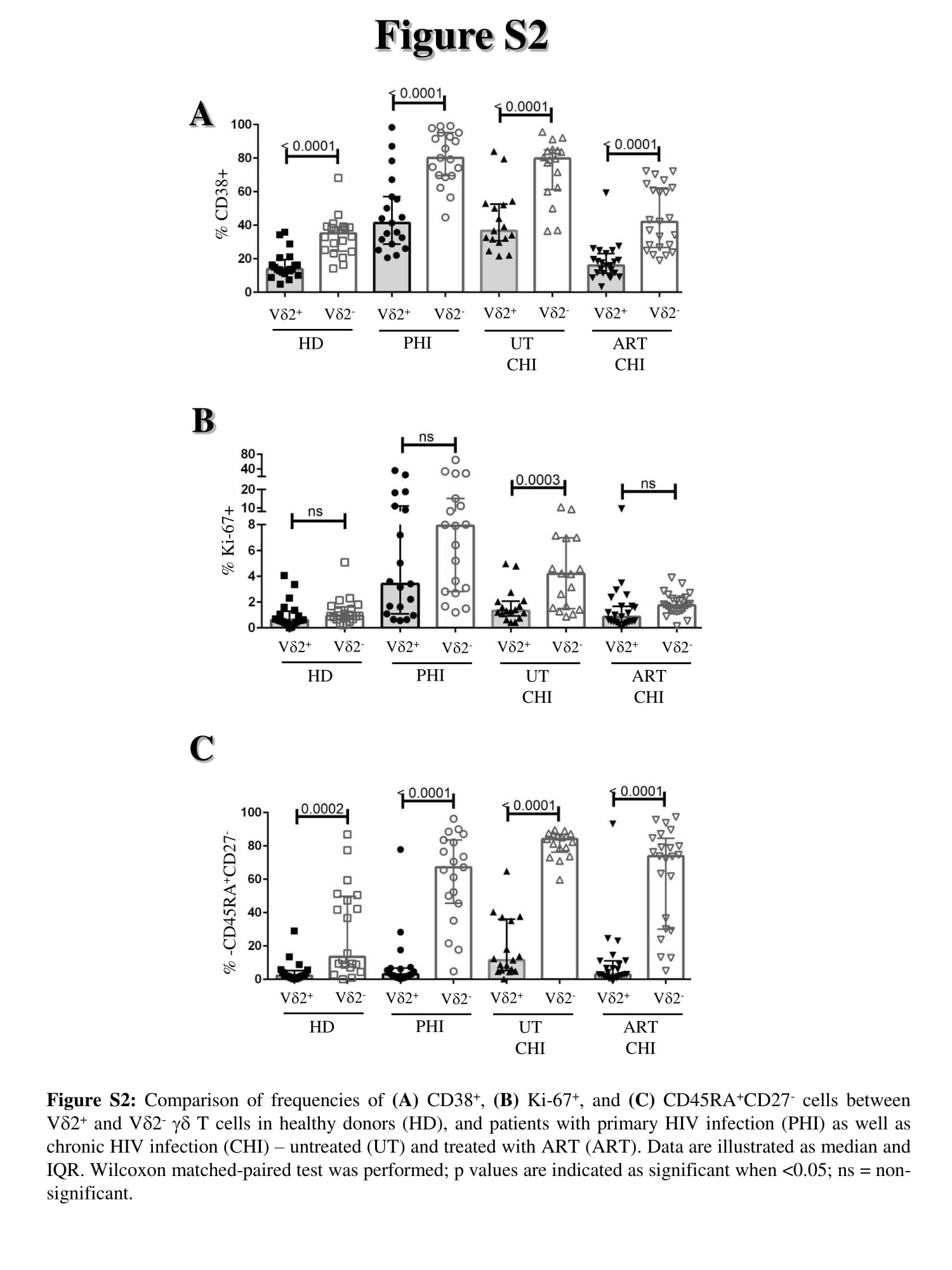

Supplement: Supplementary file 2 [file image_2.jpeg]

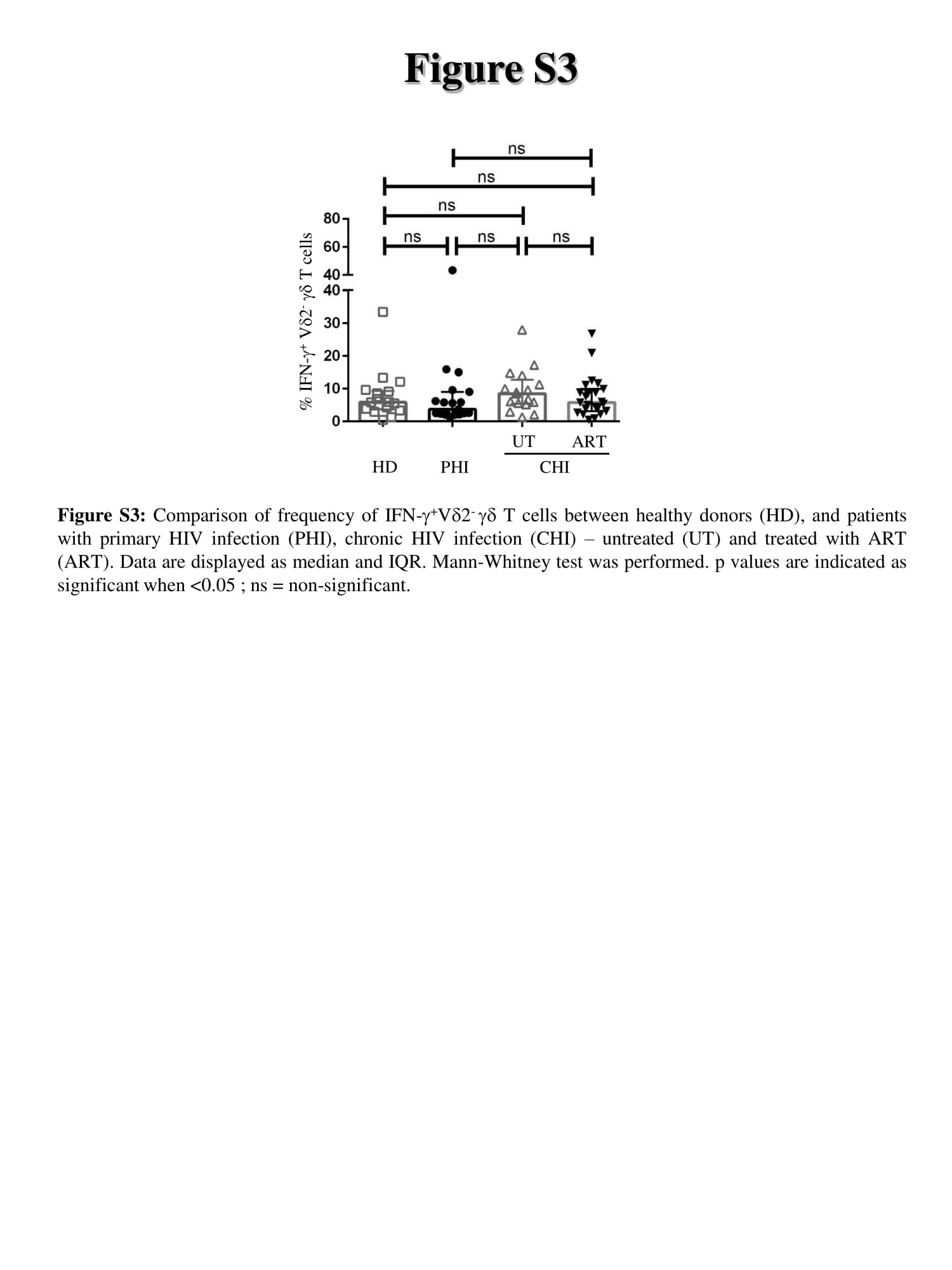

Supplement: Supplementary file 3 [file image_3.jpeg]
